# Dry Eye Para-Inflammation Management: Preclinical and Clinical Evidence on a Novel 0.2% Hyaluronic Acid-Based Tear Substitute with 0.001% Hydrocortisone Sodium Phosphate

**DOI:** 10.3390/jcm13185639

**Published:** 2024-09-23

**Authors:** Anna Rita Blanco, Giuseppe Zasa

**Affiliations:** Alfa Intes Industria Terapeutica Splendore S.r.l, Casoria, 80026 Naples, Italy; giuseppe.zasa@alfaintes.it

**Keywords:** dry eye disease, para-inflammation, hydrocortisone, artificial tears, tear substitutes, ocular surface

## Abstract

**Purpose:** An innovative eyedrop formulation based on a combination of 0.2% hyaluronic acid and 0.001% hydrocortisone sodium phosphate (Idroflog^®^, Alfa Intes, Italy; HAC eyedrops) was granted a European Patent in 2016 and has been available on the market since 2019 in Europe and in other countries around the world. HAC eyedrops aim to synergize the moisturizing effects of hyaluronic acid with the mild anti-inflammatory properties of low-dose hydrocortisone, offering a more effective and safer alternative for treating dry eye disease (DED), targeting both tear film instability and dysfunctional para-inflammation. The activity of HAC eyedrops has been explored in different post-marketing clinical trials, in addition to preclinical studies. In this narrative review, we explored the available evidence on the use of HAC eyedrops for the management of para-inflammation in DED patients to provide a comprehensive overview of efficacy and safety data related to the use of this medical device in routine clinical practice. **Methods:** A literature search for preclinical and clinical data involving treatment with HAC eyedrops was conducted using PubMed/MEDLINE, considering only original research articles published in English, without time restrictions. **Results:** One preclinical and four clinical papers were retrieved. Preclinical evidence suggests that 0.001% hydrocortisone is able to control the expression of inflammatory markers, and this, together with the hydrating and lubricating properties of hyaluronic acid, leads to improvements in DED clinical signs, such as tear volume and the stability of the tear film. The results of clinical trials demonstrate that HAC eyedrops are able to improve the signs and symptoms of DED and that 0.001% low-dosage hydrocortisone can be helpful in preventing the progression to chronic stages of DED. **Conclusions:** HAC eyedrops represent a promising therapeutic strategy for the management of dysfunctional para-inflammation and offer a valuable addition to the armamentarium of treatments for DED.

## 1. Introduction

Dry eye disease (DED) is a multifactorial ocular condition that affects millions of individuals worldwide, posing significant challenges to ocular health and the overall quality of life [1,2]. Characterized by a complex interplay of environmental, anatomical, physiological, and inflammatory factors, DED manifests through a variety of symptoms, ranging from ocular discomfort to visual disturbances, impacting daily activities and productivity [3,4]. Major risk factors for DED include female sex, older age, postmenopausal estrogen therapy or ocular surface surgery, and using antihistamine medications [5]. Moreover, the use of visual display terminal use was related to the progression of DED, which could be explained by a decreased blink rate and an increased proportion of incomplete blinks. Sunlight, outdoor environments, and air pollution have also been associated with an elevated DED risk, as well as vitamin D deficiency and diabetes mellitus [6,7]. Despite its impact and increasing prevalence, reported to be nearly one in five adults, the pathophysiology of DED remains incompletely understood, presenting a pressing need for comprehensive research and therapeutic advancements [8].

The ocular surface represents a dynamic interface between the external environment and the delicate structures of the eye, facilitated by a precise balance of tear film components. The disruption of this delicate equilibrium, whether due to aqueous deficiency, evaporative factors, or a combination thereof, leads to the hallmark signs and symptoms of DED [9]. While traditional paradigms focused primarily on tear quantity and quality, recent insights underscore the crucial role of ocular surface inflammation in the pathogenesis of DED [10]. Indeed, DED begins with tear film instability and hyperosmolarity, which damage the ocular surface and trigger the release of proinflammatory cytokines. These cytokines attract immune cells, leading to chronic inflammation that perpetuates the cycle of tear film disruption and ocular surface damage. This ongoing inflammatory process not only exacerbates the symptoms of DED, such as discomfort and pain, but also contributes to the chronic nature of the disease, establishing a vicious circle [4].

It is noteworthy that before the vicious circle underlying DED pathogenesis is triggered, a transient para-inflammatory phase is established as a mechanism that can restore ocular surface homeostasis [11]. In detail, para-inflammation in the context of DED refers to a low-level, adaptive response that is triggered by cellular stress on the ocular surface, particularly when the tissue is exposed to environmental or physiological challenges that are insufficient to cause full-blown inflammation. It serves as a protective mechanism, aiming to restore homeostasis and maintain tissue function [11]. Accordingly, para-inflammation has been defined as a biphasic dose–response, where low doses of stimuli result in protective effects that can lead to improved organism performance [12]. However, in the presence of recurrent or persistent external stimuli, this regulatory mechanism becomes dysfunctional and contributes to the development of the disease, whose severity is related to the nature and intensity of the external challenge. This chronic state may lead to tissue damage and contribute to the worsening of DED, overcoming the line between adaptive protection and harmful inflammation [13]. Consequently, dysfunctional para-inflammation is now recognized as the starting point for the development of a stable, chronic inflammatory disease, which leads to ocular surface damage and, in turn, to the onset or progression of chronic DED [13].

Recent observations highlight that tear cortisol is significantly reduced in dry eye patients [14]. As an endogenous glucocorticoid produced in small amounts by corneal cells, cortisol has immunomodulatory effects on the ocular surface [15,16,17,18]. The 11ß-Hydroxysteroid dehydrogenase type 1 (11 β-HSD-1) enzyme, which catalyzes the conversion of cortisone to cortisol, has been found in the basal cells of the corneal epithelium [19], suggesting that, even in this district, cortisol may act to keep the immune response repressed and help the system recover its homeostasis. In line with this hypothesis, recent studies have shown that the supplementation of tear cortisol with the instillation of a very low, sub-pharmacological dose of hydrocortisone, a lipophilic analog of cortisol with low anti-inflammatory properties and a short duration of action, effectively modulated proinflammatory cytokines on the ocular surface and improved tear film stability and production [16].

Artificial tears (ATs) are considered the first-line management option for DED, as they are easy to use, accessible in a wide range of formulations, and have a low-risk profile [19]. Accordingly, a multitude of tear substitutes are currently available on the market worldwide, with a wide variety of ingredients [20]. Most of these ATs are classified as medical devices (MDs) and contain a wide variety of ingredients (e.g., viscosity-enhancing agents, electrolytes, osmo-protectants, antioxidants, lipids, surfactants, and preservatives); however, few studies have been performed to better understand the specific role of each ingredient composing the different formulations, and there is inconsistency in clinical trials assessing the safety and performance of a lot of these tear substitutes [20].

Among viscous polymers, one clinically proven and commonly used is hyaluronic acid (HA), a naturally occurring and non-toxic glycosaminoglycan. Due to its safety profile and physiological effects, it has become an important molecule in ophthalmology [21]. It has appropriate rheological features and binds water molecules, thickening and stabilizing the tear film, reducing the effects of mechanical trauma to the ocular surface by lubrication, and contributing to re-epithelization. Moreover, HA reduces evaporation from the ocular surface, which is one of the driving forces behind hyperosmolarity at the basis of DED pathogenesis [22].

**Figure 1:**
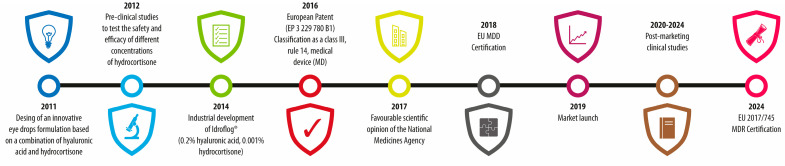
Main development steps of HAC eyedrops. EU MDD: European Medical Device Directive; EU MDR: European Medical Device Regulation.

Accordingly, HA was the starting point for the development of an innovative eyedrop formulation based on a combination of 0.2% HA and 0.001% hydrocortisone sodium phosphate (Idroflog^®^, Alfa Intes, Casoria, Italy; hereafter termed HAC eyedrops), which was granted a European Patent in 2016 (EP 3 229 780 B1) [23] and has been available on the market since 2019 (Figure 1) in Italy and in some other European and non-European countries. This innovative formulation, which is available as single-dose and preservative-free bottle eyedrops, aims to synergize the moisturizing effects of hyaluronic acid with the mild anti-inflammatory properties of low-dose hydrocortisone, offering a more effective and safer alternative for treating DED, targeting both tear film instability and dysfunctional para-inflammation while minimizing potential side effects.

HAC eyedrops are classified as a class III, rule 14, medical device (MD) according to Regulation (EU) 2017/745 (MDR). This Class of Medical Device (Class III, Rule 14) requires the execution of clinical trials to provide a better understanding of the properties of the different types of tear substitutes and, more specifically, the roles of their components. That could help physicians determine the most suitable tear substitute for each DED patient, in line with the clinical findings and DED features. According to this new regulation on MDs, the activity of HAC eyedrops has been explored in different post-marketing clinical trials, in addition to preclinical studies conducted to obtain a CE mark. In this narrative review, we explored the available preclinical and clinical evidence on the use of HAC eyedrops for the management of para-inflammation in DED patients to provide a comprehensive overview of efficacy and safety data related to the use of this medical device in routine clinical practice.

## 2. Methods

A literature search for preclinical and clinical data involving treatment with HAC eyedrops was conducted considering only original research articles published in the English language without time restrictions, up to June 2024. The search terms used in PubMed/MEDLINE included “hydrocortisone AND dry eye disease”, “hydrocortisone AND para-inflammation”, “Idroflog AND dry eye disease”, and “Idroflog AND para-inflammation”. The references of the articles were screened based on titles and abstracts to identify relevant papers on these topics. Further full-text screening was carried out to identify gaps in the selected literature and elaborate on this topic comprehensively.

## 3. Results

Overall, one preclinical paper and four clinical papers were retrieved by the literature search. A summary description of the study results is provided in the following paragraphs.

### 3.1. Preclinical Evidence on Pharmacological Profile of Hydrocortisone in Dry Eye Disease

Bucolo et al. investigated the ocular pharmacological profile of hydrocortisone (HC) using in vitro and in vivo models of DED [16]. Rabbit corneal epithelial cells (SIRCs) were used to assess the effect of HC in two paradigms of corneal damage: hyperosmotic stress and the scratch-wound assay; DED was induced in albino rabbits by the administration of atropine sulfate into the lower conjunctival sac of the eye (four times within 12 h), which is able to decrease tear volume and alter tear film stability thanks to its activity as a muscarinic (M3) receptor antagonist. Another set of rabbits was treated with the T-cell mitogen concavalin A (ConA) in the lacrimal glands using a 28-gauge needle [16].

In the in vitro assessments, they found that rabbit corneal epithelial cells exposed to a 24 h hyperosmotic insult showed significant increases in TNFα, IL-1β, and IL-8 levels (*p* < 0.05) that were significantly counteracted by 0.001% HC treatment (*p* < 0.05). The 0.001% HC treatment was also able to significantly increase (*p* < 0.05) TNF-related apoptosis-inducing ligand (TRAIL) expression, recognized as a protective factor [24], compared to cells exposed to hyperosmotic stress.

In rabbit models, this study demonstrated that HC crossed, in a dose-dependent manner, the corneal barrier when the eyes were topically treated with four formulations containing different concentrations of hydrocortisone (range 0.001–0.33%) and 0.2% hyaluronic acid, administered four times every 2 h to provide a sustained treatment [16]. However, no levels of HC were detected in the aqueous humor after the topical administration of the lowest HC dose (0.001%; HAC eyedrops) among the tested formulations, suggesting that, at this very low concentration, the drug did not cross the corneal barrier, avoiding potential side effects such as an intraocular pressure rise [16]. In atropine DED rabbit models, HAC eyedrops significantly restored the tear volume and tear film integrity to the levels of the control eyes, showing no change in IOP values throughout the treatment period. Moreover, HAC eyedrops significantly reduced the tear levels of TNF-α, IL-8, and MMP-9 when compared with the vehicle in the ConA DED model. Again, in this model, the HAC eyedrop treatment significantly counteracted the tear volume reduction and restored tear integrity (*p* < 0.05) [16]. Altogether, these data suggest that HC, at very low concentrations, has an important anti-inflammatory effect in both in vitro and in vivo dry eye paradigms and a good safety profile.

### 3.2. Clinical Evidence on the Activity and Safety of HAC Eyedrops for the Management of DED

The double-blind, single-center, randomized clinical trial conducted by Cagini and collaborators was designed to assess the kinetics of hydrocortisone sodium phosphate penetration into the human aqueous humor after topical application [25]. Patients who had undergone phacoemulsification with intraocular lens implantation were randomly assigned on the morning of surgery to receive a single instillation of one of two products containing hydrocortisone at different concentrations: Cortivis^®^ 0.33% (drug, group 1) or Idroflog^®^ 0.001% (HAC eyedrops, class III MD, group 2). A third group of patients did not receive any treatment and was used to measure endogenous hydrocortisone levels. Immediately before surgery, an aliquot of aqueous humor was aspirated by means of paracentesis using an insulin syringe. The aqueous humor sample was promptly transferred to Eppendorf vials and immediately stored at −20 °C. Hydrocortisone concentrations were obtained by liquid chromatography–mass spectrometry (LC/MS) analysis; hydrocortisone and D4-hydrocortisone were identified as [M-H]− ions based on accurate mass and MS/MS spectra [25]. The mean concentration of HC measured in aliquots of aqueous humor was higher in group 1 patients (25.2 ± 12.4 ng/mL) compared with group 2 (7.11 ± 1.51 ng/mL) and compared to the mean HC endogenous levels (3.92 ± 1.18 ng/mL) (*p* < 0.0001) detected in untreated control patients. No significant differences between the mean HC concentration in group 2 and the mean endogenous HC level were found [25]. This study suggests that, considering the frequent need for prolonged topical steroid therapies and the possible consequent undesirable side effects, ophthalmologists should consider the lowest clinically effective dose of hydrocortisone useful to obtain the desired therapeutic effect and at an adequate time to minimize the level of steroids in the anterior chamber and to avoid side effects like an intraocular pressure increase or cataract development.

A randomized, controlled, double-masked study was conducted to evaluate the long-term (up to 6 months) activity and safety of HAC eyedrops in 40 patients with DED [26]. The treatment with HAC eyedrops was compared with a control 0.2% HA ophthalmic solution after an initial 7-day treatment with corticosteroid (fluorometholone eyedrop). This standard corticosteroid treatment aimed to bring all patients to a comparable baseline condition (para-inflammation), from which the performance of the two artificial tears could be better evaluated. Significant improvements in the frequency and intensity of DED symptoms were observed in both groups, but after corticosteroid discontinuation, the maintenance of the therapeutic advantage was observed only in the HAC eyedrop group, which also showed a significant reduction in the tear film break-up time (*p* ≤ 0.05) and infiltrated macrophages (*p* < 0.05) after only 1 month of treatment, which was assessed as the number of infiltrating macrophages (CD14+) compared with the number of total leukocytes (CD45+; reported as CD14+/CD45+ ratio). This suggests that the HAC eyedrops are able to control macrophage infiltration better than the control treatment, prolonging the control of the immune system involvement and, through para-inflammation, leading to an easier recovery of the homeostasis condition [26]. A significant reduction in fluorescein and Lissamine staining (*p* < 0.05) was also observed in the HAC eyedrop group, suggesting damage reduction at both corneal and conjunctival levels [26]. Intraocular pressure was maintained within the normal range for both groups, supporting the product’s safety even in the case of long-term treatment [26]. According to the collected evidence, the use of HAC, even in the initial stages of DED, can be suggested to achieve the rebalance of ocular surface alterations, preventing degeneration toward a pathological condition that could become chronic. Furthermore, the product’s safety has been observed even after continuous use, allowing a prolonged treatment [26].

In a prospective randomized study, 38 patients with moderate DED were divided to receive one drop of 0.2% sodium hyaluronate and 0.001% HC (HAC eyedrops) four times daily for 3 months or 0.15% sodium hyaluronate and 3% trehalose at the same dosage [27]. Patients treated with HAC eyedrops showed significant improvement in all the assessed signs and symptoms of DED, while in the trehalose group, a significant improvement was observed only in the TBUT score (*p* < 0.05). A more in-depth evaluation of both groups highlighted a different and significant effect of HAC eyedrops on the non-invasive break-up time (NIKBUT) and lipid layer thickness (LLT) at the end of treatment (*p* = 0.001 for both comparisons) [27]. This evidence supported the effectiveness and safety of HAC eyedrops in the treatment of moderate/severe DED over a 3-month period, showing the ability to restore ocular surface homeostasis by exploiting cortisol activity to regulate para-inflammation, in addition to the conventional moisturizing effect of HA [27].

Lastly, a very recent study assessed the effectiveness of HAC eyedrops in treating the signs and symptoms of DED in comparison with standard tear substitutes (STSs) by means of low- and high-tech assessments [28]. DED diagnosis was related to post-cataract surgery, meibomian gland dysfunction (MGD), allergy, or glaucoma medications. The obtained results highlighted the effectiveness of HAC eyedrops in all DED subtypes, as well as its superiority over STSs in terms of tear film stability improvement when changes were inspected by NIKBUT-first [28]. In particular, NIKBUT-first in the HAC eyedrop group was enhanced in patients with MGD after 15 days of treatment and in patients who had undergone cataract surgery after 45 days of treatment. Moreover, only patients in the HAC eyedrop group reported a statistically significant improvement in the NIKBUT class after 45 days of treatment [28]. In this study, although both the tear meniscus height (TMH) and the Schirmer test indicated significant improvement related to the tear quantity at follow-up, the TMH was able to identify earlier and more frequent changes than the Schirmer test, which were all in favor of the HAC eyedrop group. Moreover, when considering hyperemia in patients with DED after cataract surgery, the improvement was significant after treatment with HAC eyedrops compared with the baseline, although without a difference from STSs [28]. Longer observation periods (i.e., 3–6 months) were recommended to fully ascertain whether the early improvement detected by high-tech measures can be confirmed at subsequent time points, even when using low-tech tests [28].

## 4. Concluding Remarks

The effective management of para-inflammation in DED is crucial for preventing the transition from a protective response to harmful chronic inflammation. In particular, early intervention to control para-inflammation can reduce tissue damage, alleviate symptoms, and slow the progression of DED, improving patient outcomes and preserving long-term eye health.

**Figure 2:**
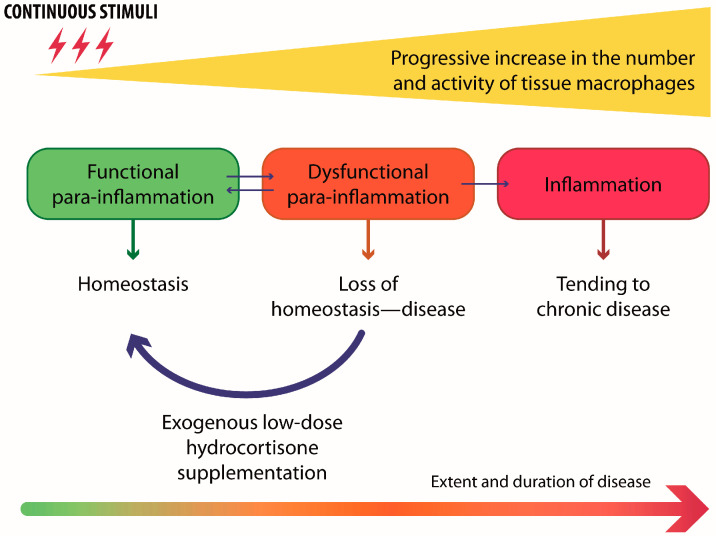
A schematic representation showing the process that leads from the homeostasis of the ocular surface to chronic disease and the potential role of exogenous hydrocortisone supplementation to restore homeostasis in the case of dysfunctional para-inflammation. Adapted from [26], published as an open-access article distributed under the terms and conditions of the Creative Commons Attribution (CC BY) license.

Artificial tears are considered the mainstay treatment of DED [19]. HA-based tear substitutes have gained popularity due to their ability to provide lubrication and hydration to the ocular surface. Furthermore, the addition of low-dose HC (0.001%) to sodium hyaluronate within an innovative eyedrop formulation (HAC eyedrop, class III MD) showed the potentiality to modulate dysfunctional para-inflammation without side effects (Figure 2) [26,27,28].

Preclinical evidence from in vitro and in vivo DED models suggests that HC, at very low concentrations (0.001%), is able to control the expression of inflammatory markers, and this, together with the hydrating and lubricating properties of HA, leads to an improvement in DED clinical signs, such as the tear volume and the stability of the tear film [16]. A good safety profile was also reported, considering that the 0.001% HC dose was shown to not cross the corneal barrier, thus avoiding potential side effects [16]. This is further supported by the observation that the aqueous humor’s mean HC concentrations were not increased after the instillation of 0.001% HC when compared to endogenous cortisol levels, as observed in a group of patients who had undergone phacoemulsification with intraocular lens implantation [25,26].

The results of clinical trials demonstrate that the innovative eyedrop formulation based on 0.2% hyaluronic acid and 0.001% hydrocortisone (HAC eyedrops) is able to improve the signs and symptoms of DED and that 0.001% low-dosage HC can be helpful in preventing the progression to chronic stages of DED [27,28]. In particular, when compared with the use of standard substitutes, HAC eyedrops showed persistent and long-term activity and seemed to counteract macrophage infiltration, prolonging the control of the immune system involvement and, through para-inflammation, leading to an easier recovery of homeostatic conditions [26]. The superiority of HAC eyedrops over STSs in terms of tear film stability improvement was also reported after an assessment with high-tech parameters (NIKBUT-first). It was shown to be enhanced in specific subgroups of patients, such as those who had undergone cataract surgery [28]. Moreover, safety data from clinical trials also indicate the stability of the IOP parameter during the long-term use of HAC eyedrops, correlating with data reporting the stability of mean HC concentrations in the aqueous humor [25,26].

An appropriate Post-Marketing Surveillance System constantly monitors the Performance and Safety profile of the product. It is interesting to note that HAC eyedrops have been on the EU market for 5 years; to date, there have been no incidents and/or adverse events that could compromise the health and safety of the patient/user.

In conclusion, HAC eyedrops represent a promising therapeutic strategy for the management of para-inflammation and offer a valuable addition to the armamentarium of treatments for DED, addressing the underlying inflammatory component of the disease while providing symptomatic relief and improving patient outcomes. Further research is warranted to better understand the precise role of endogenous tear cortisol and whether low concentrations of exogenous hydrocortisone supplementation can actually bring cortisol tear levels to physiological values, helping the system to recover a state of homeostasis, also better controlling the inflammatory spikes that characterize DED. Overall, these observations can support and further explain the positive clinical results on the effects of HAC eyedrops on the signs and symptoms of DED.

## Data Availability

No new data were created or analyzed in this study. Data sharing is not applicable to this article.
